# Model ensembling as a tool to form interpretable multi-omic predictors of cancer pharmacosensitivity

**DOI:** 10.1093/bib/bbae567

**Published:** 2024-11-04

**Authors:** Sébastien De Landtsheer, Apurva Badkas, Dagmar Kulms, Thomas Sauter

**Affiliations:** Department of Life Sciences and Medicine, University of Luxembourg, 2, place de l’Université, L4365 Esch-sur-Alzette, Luxembourg; Department of Life Sciences and Medicine, University of Luxembourg, 2, place de l’Université, L4365 Esch-sur-Alzette, Luxembourg; Experimental Dermatology, Department of Dermatology, Technische Universität-Dresden, 01307 Dresden, Germany; National Center for Tumor Diseases, Technische Universität-Dresden, 01307 Dresden, Germany; Department of Life Sciences and Medicine, University of Luxembourg, 2, place de l’Université, L4365 Esch-sur-Alzette, Luxembourg

**Keywords:** cancer, pharmacosensitivity, machine-learning, predictive algorithm, CCLE

## Abstract

Stratification of patients diagnosed with cancer has become a major goal in personalized oncology. One important aspect is the accurate prediction of the response to various drugs. It is expected that the molecular characteristics of the cancer cells contain enough information to retrieve specific signatures, allowing for accurate predictions based solely on these multi-omic data. Ideally, these predictions should be explainable to clinicians, in order to be integrated in the patients care. We propose a machine-learning framework based on ensemble learning to integrate multi-omic data and predict sensitivity to an array of commonly used and experimental compounds, including chemotoxic compounds and targeted kinase inhibitors. We trained a set of classifiers on the different parts of our dataset to produce omic-specific signatures, then trained a random forest classifier on these signatures to predict drug responsiveness. We used the Cancer Cell Line Encyclopedia dataset, comprising multi-omic and drug sensitivity measurements for hundreds of cell lines, to build the predictive models, and validated the results using nested cross-validation. Our results show good performance for several compounds (Area under the Receiver-Operating Curve >79%) across the most frequent cancer types. Furthermore, the simplicity of our approach allows to examine which omic layers have a greater importance in the models and identify new putative markers of drug responsiveness. We propose several models based on small subsets of transcriptional markers with the potential to become useful tools in personalized oncology, paving the way for clinicians to use the molecular characteristics of the tumors to predict sensitivity to therapeutic compounds.

## Graphical Abstract

**Graphical Abstract:**
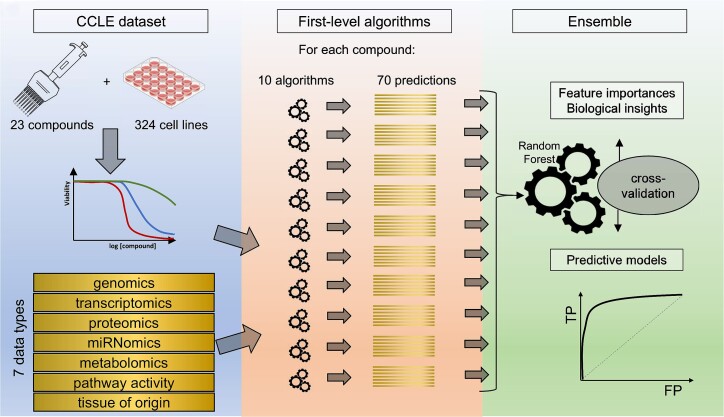

## Introduction

Despite major breakthroughs in targeted tumor treatment options over the past few decades, cancer remains the second leading cause of deaths worldwide [1]. One of the reasons for this is the fact that intervention strategies are exclusively based on the mutation status of key oncogenic drivers of a specific tumor type. However, tumors present with high heterogeneity, even within a certain tissue, and despite similar clinical features. The degree of heterogeneity itself is highly variable: a number of hematological malignancies are defined by precise chromosomal alterations, for example the reciprocal translocation t(9;22)(q34;q11) resulting in the chimeric BCR-ABL protein in virtually all cases of chronic myeloid leukemia [2]. In contrast many different driver mutations are implicated in the most common tumor types, especially melanoma [3] and lung adenocarcinoma [4]. Above this, tumor heterogeneity is constantly reinforced by the fact that most tumors are deficient in proper deoxyribonucleic acid (DNA) repair, thereby further increasing their mutational load. The cancer hallmarks [5], a set of phenotypic capabilities shared by all tumors and central to their emergence and evolution toward malignancy, have been shown to be highly polygenic, while the main cancer genes are pleiotropic [6], and are found to be mutated across tumor types. For example loss- or gain-of function mutations of the transcription factor p53 (TP53) occur in ~50% of all human cancers [7]. Moreover activating mutations of the mitogen-activated kinase BRAF can be found across a variety of cancers, including melanoma, colon adenocarcinoma, and glioma [8]. While dozens of chemotherapeutics, cytotoxic or targeted compounds have been approved for cancer treatment over the past decades, they will only be efficacious in a subset of cancer patients, mainly because additional pathophysiological modifications, involving differential expression of genes/proteins within the oncogenic signal transduction network may contribute to therapy resistance.

Subsequent to the identification of druggable molecules within this network, targeted therapeutics were designed to interfere with a specific protein, either via a small compound, like tyrosine kinase inhibitors, or a specific antibody [9, 10]. Despite the increasing knowledge on cancer-specific signal transduction and the development of targeted drugs, initial response rates of patients remain low, or they may quickly acquire resistance [11]. It is therefore essential to expand the arsenal of stratification tools to better identify tailored drug regimens to increase response rates and decrease unnecessary treatment burden and side effects for cancer patients. Ultimately, the goal of personalized oncology is to be able to treat each cancer patient based on the unique array of characteristics of their tumors, and in the context of their germline genomes and clinical histories.

In order to capture the multiple layers of the regulatory network ultimately contributing to cancer development and progression, large-scale screenings have been performed to characterize panels of cell lines across multiple omics levels, together with measurements of drug responsiveness. The Cancer Cell Line Encyclopedia (CCLE) dataset [12] presents the most prominent results of such screening efforts, containing data on more than a thousand cell lines of various cancer types and subtypes including high-quality multi-omic data and pharmacological characterization, and has been shown to enable predictive modeling of drug responsiveness [12].

Key points in the application of artificial intelligence to precision oncology have been highlighted elsewhere in excellent reviews [13–15]. The NCI-60 cell line panel pioneered the use of a large screening to discover characteristics of cell lines indicative of chemosensitivity [16]. Modeling was first applied to the problem of predicting cell line chemosensitivity by Staunton *et al.* [17], originally a simple weighted voting scheme. Later, a genetic signature based on the expression of 70 marker genes was used to predict the clinical outcome of breast cancer patients [18]. Mathematical modeling was then extended to various frameworks, notably the use of kernel methods [19], regularized linear regressions, such as the Elastic Net or the LASSO [20], regression, and classification trees [21], matrix factorization [22], then to various neural-networks-based algorithms like Deep Learning [23–25] and Graph Convolutional Networks [26, 27]. A number of studies included the chemical structure of compounds as a component of their models [28, 29]. In addition, a number of interesting studies have investigated the application of multi-omic models to predict the effect of drugs, including side effects [30–32]. Recent efforts to integrate multiple omic types in a modified deep-learning framework comprise TMO-Net [33] and AutoSurv [34]. Despite continuous improvements, predictions formed with simple, interpretable methods usually fail to reach validation in a clinical setting, and the best performing pre-clinical methods, often composed of complex black-box algorithms, lack interpretability.

Notably, the NCI-DREAM challenge [35], which compared the predictions of 44 teams for a breast-cancer sensitivity prediction task, concluded that differences in performance between the algorithms can mostly be attributed to data quality, preprocessing strategies, and choice of the reported variable, rather than the family of the method used. It also clarified that predictions based on the combinations of individual teams’ algorithms always outcompeted the best of the individual methods, showing that different methods provide complementary information.

Therefore, a method to combine predictions of the various methods is needed. Stacking [36] is an ensemble learning technique that first trains a series of classifiers on labeled training data, then trains a second-level generalizer aiming to learn the biases of the individual classifiers with respect to the true labels of the training set. Stacked ensembles have been shown to lower the predictor bias and, in any case, produce results that are no worse than the best individual model [37].

In this paper, we hypothesize that while each individual omic type contains only a partial signal, it is possible to combine the imperfect information gathered from each biological layer into an integrated picture of the particular tumor and deduce the drug-resistant *versus* drug-sensitive profile. We also hypothesize that once a ‘black-box’ model is established, it is possible to retrieve the most important sources of predictive signals, combine them in a top-down manner, to engineer an explainable interpretable model, which could be evaluated in a clinical setting in the future. Importantly, we assume that while heterogeneity between patients, and therefore between cell lines, is large, homologies can be extracted given a large enough sample size, allowing to learn robust correlations between molecular and functional states.

## Methods

### Data source

CCLE data files were downloaded directly from the DepMap portal (https://depmap.org/portal/). For transcriptomics, we used the provided file *CCLE_RNAseq_genes_rpkm_20180929.gct* containing rpkm values (reads per kilobase per million reads mapped) for 56 202 transcripts. We did not aggregate the data at the gene level to allow for discovery of splice variants associated with functional response. For genomics, we used the file *CCLE_MUT_CNA_AMP_DEL_binary_Revealer.csv* summarizing the presence versus absence of specific genetic features for all cell lines as a Boolean table. For the micro ribonucleic acid (miRNA), we used the file *CCLE_miRNA_20181103.csv* containing fpkm (fragments per kilobase of transcript per million fragments mapped) values for 974 miRNAs. The metabolomics data consisted of profiles for 225 metabolites, determined by Liquid Chromatography Mass Spectrometry (LS-MS) in the file *CCLE_metabolomics_20190502.csv*. For the proteomics data, we used the file *CCLE_RPPA_20181003.csv* consisting of reverse-phase protein array (RPPA) measurements of 214 proteins including protein modifications. In addition, we included the estimates of pathway activity found in the file *1-s2.0-S0092867416307462-mmc6.xlsx* from the GDSC study [38] for the samples included in both GDCS and CCLE databases. These pathway activities were pre-computed from gene expression using the algorithm SPEED [39].

The 23 drugs studied in this paper are AEW541, nilotinib, 17-AAG, PHA-665752, lapatinib, nutlin-3, AZD0530, PF2341066, L-685458, ZD-6474, panobinostat, sorafenib, topotecan, LBW242, PD-0325901, PD-0332991, paclitaxel, AZD6244, PLX4720, RAF265, TAE684, TKI258, and erlotinib.

### Preprocessing

Table S1 describes the filtering steps that were applied to each dataset. Briefly, quantitative data was log-transformed and normalized to the [0, 1] interval to facilitate modeling. We avoided the need for data imputation by removing samples and features with missing data. Then, we applied a simple feature selection scheme, by first removing a proportion of features showing low variance across the samples, and subsequently removing highly cross-correlated features. We extracted cancer type (tissue of origin) for each sample from the samples’ names. The pre-processed dataset used in following steps contained a total of 324 samples from 23 different cancer types, and 48 453 features. Drug response information, in the form of the ActArea (normalized area over the drug-response curve, a proxy for cell line sensitivity which takes partial response into account, in contrast with the IC_50_) was collected for the 23 compounds (topotecan was removed from the dataset as data for this drug was incomplete) and quantized into three categories: resistant (one-third of cell lines with the smallest ActArea), sensitive (one-third of cell lines with the largest ActArea), and intermediate. This latter stratum was excluded from subsequent modeling steps, to exaggerate the differences between resistant and sensitive cell lines and to avoid mislabeling. While this drug-agnostic labeling might not be the most appropriate for all compounds and may not accurately reflect the levels of drug responsiveness of samples in a clinical context, it has the advantage of framing the study as a simple binary classification problem on a balanced dataset, thus avoiding the need for multi-class models, over/undersampling and data augmentation, which would possibly induce more serious biases on the methodology and the interpretation of the results.

### Stacking methodology

**Figure 1:**
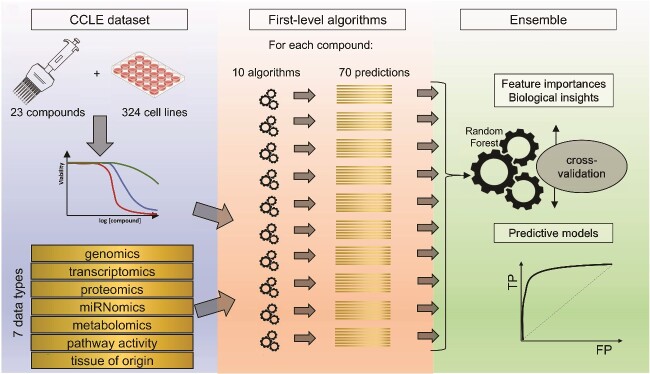
Schematized procedure. The CCLE dataset preprocessing and filtering resulted in a data matrix of 23 anti-cancer compounds and 324 cell lines, with dose-response curves for each combination, and multiomic data for each cell line. 10 different machine-learning algorithms were trained to predict sensitivity separately on each omic type, resulting in 70 predictions. The final prediction is made by an ensemble random forest integrating the predictions of the first-level algorithms. This ensemble model is assessed by cross-validation and examined for the importance of individual features.

The following nested cross-validation procedure was used to build the classifiers for each drug. In the first step, the dataset was split into a ‘training’ set (90% of samples) and a ‘test’ set (10% of samples). The ‘training’ set was then split further into a ‘training A’ set (81% of samples) and ‘training B’ (9% of samples). Then, first-level algorithms (see Supplementary methods) were trained independently on the ‘training A’ set of samples, using in turn each one of the seven omic layers, to form a prediction of the probability of class membership (sensitive or resistant) of each sample. These trained models were then used to predict the class of the samples in the ‘training B’ set. This procedure was repeated over 10 non-overlapping splits of the ‘training’ set, producing quantitative predictions for each sample in the ‘training’ set, as well as for the ‘test’ set (using in that case algorithms trained on the whole ‘training’ set). These probabilities of class memberships were then used to train a second-level random forest: using the ‘training’ predictions (cumulated over the 10 splits) to form a combined prediction of class membership for the samples in the ‘test’ set, therefore using predictions formed on all omic layers. This complete procedure was repeated 10 times in order to produce a final prediction for every sample in the dataset while avoiding data leakage. The procedure is illustrated in Fig. 1.

### Explainable models

Drawing from the previous analyses and to propose clinically applicable tools, we built simple predictive models. For each drug, we focused on the transcriptomic data, and we restricted the number of predictors to the top three genes showing the highest importance in previous analyses. The selected genes for each drug are compiled in Supplementary Table S2. Furthermore, we only considered three types of models, chosen for their simplicity of interpretation: linear regression, logistic regression, and single decision tree. The magnitude of the coefficients of the regression models and the structure of the tree can be interpreted biologically in a straightforward manner. We trained the three model types independently for each drug and selected the model with the largest area under the receiver operating curve (AUROC). To test our models on another sample set than the one from which the features were selected, we recovered the samples that were excluded at the beginning of the analysis because one of the data types (usually the proteomics) was absent. In total, we recovered 695 samples with both drug and transcriptomic information.

## Results

### Ensembles can predict sensitivity versus resistance for both cytotoxic and targeted drugs

In this study, we sought to evaluate the performance of stacked classifiers (random forests) for the task of discriminating the most sensitive cell lines from the least sensitive ones, in the CCLE database of drug response profiles. These classifiers were based on the predictions of first-level learners (both tree-based and regression-based), trained independently on specific molecular features of the cell lines: genomics, transcriptomics including miRNomics, proteomics, metabolomics, as well as the tissue of origin and 11 pathway-level features. The complete pre-processed dataset comprised 48 453 features for 324 cell lines.

**Figure 2:**
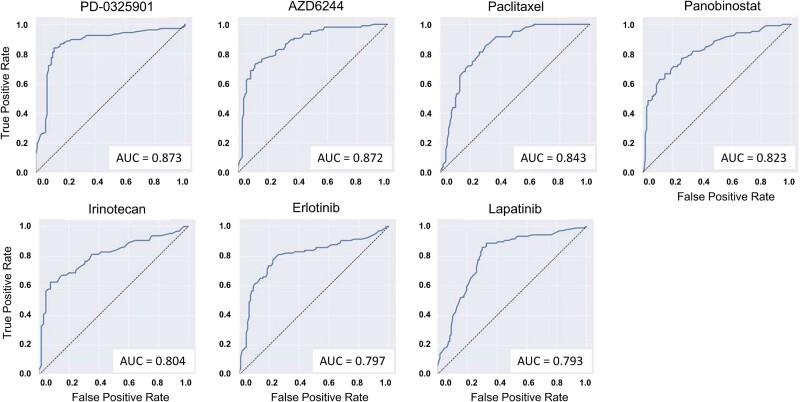
ROC curves showing the performance of the seven best predictive models. The jagged curves show the model performances as the relationship between sensitivity (true positive rate, y-axis) and specificity (false negative rate, x-axis) for different decision thresholds. The dashed line shows the theoretical performance of a random model.

We generated quantitative predictions by applying a two-step 10-fold nested cross-validation scheme and used them to compute the AUROC for each drug-specific classifier. Seven classifiers obtain an AUROC >0.75 (Fig. 2). AUROC values for the remaining 16 classifiers ranged from 0.509 to 0.721. Supplementary Fig. S1 shows the results for the complete set of 23 compounds.

**Figure 3:**
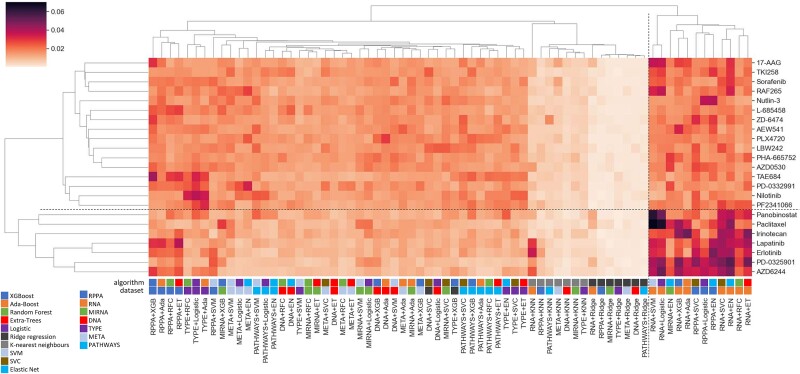
Clustergram of the average feature importance of the different combinations of omic types and predictive algorithms. The dendrograms were computed using the UPGMA algorithm and Euclidian distance. The dashed lines delimitate clusters of drugs and algorithm+datasets combinations with notable differences. RPPA: proteomics; RNA: transcriptomics; DNA: genomics; MIRNA: micro-RNAs; TYPE: cell type of origin; META: metabolomics; PATHWAYS: SPEED pathway activities; RFC: random forest classifier; ET: extra-trees classifier; XGB: XGBoost classifier; Ada: AdaBoost classifier; EN: elastic net classifier; Ridge: Ridge regression classifier; KNN: k-nearest neighbors classifier.

Furthermore, we retrieved the feature importances from the classifiers, with the hypothesis that the predictive signal in each omic type might be best recovered by certain types of algorithms, but also drug-specific. We computed the average feature importance for each combination of omic and first-level classifier across the 10-folds (Fig. 3). A clear separation between a branch containing the seven compounds for which excellent results were obtained and the others can be observed, indicating that responses to these seven compounds (panobinostat, paclitaxel, irinotecan, lapatinib, erlotinib, PD-0325901, and AZD6244) are more easily predictable. Also visible are 3 main branches of features: one containing 12 combinations of omic/algorithm with the highest contributions and enriched in transcriptomics datasets, another containing 14 combinations with very low contributions and grouping all combinations using the k-nearest neighbors and ridge regression algorithms, and a third one containing the remaining combinations with intermediate contributions. This seems to indicate that transcriptomic data carries more information that is useable by our method to predict functional responses.

### Classifier performances are tissue type-dependent

**Figure 4:**
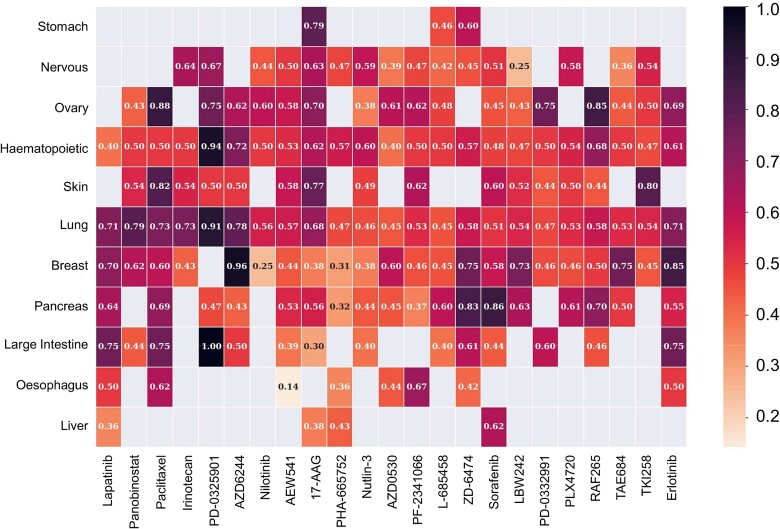
Heatmap of the BA of drug-specific predictive models against specific tumor types. BA is not reported (grey) when N < 10.

Because the cell type of origin of a tumor is nearly always known, we sought to estimate the performance of the classifiers on specific cancer types, with the two caveats that, by subsampling our balanced dataset, we introduce a degree of imbalance in the sample, and that many of the 23 cancer types are represented only by a low number of cell lines. We therefore report the balanced accuracy (BA) which is the average of specificity and sensitivity, by cell type and drug (Fig. 4). The values are omitted when the total number of cell lines is inferior to 10.

BA was found to be highly dependent of the compound and of the cell type of origin of the tumor. For example, in the case of PD-0325901 (mirdametinib, an investigational MEK inhibitor [40]), high performance was achieved in the cases of colorectal cancer (BA = 1.0 for 13 sensitive and 1 resistant cell line), lung adenocarcinoma (BA = 0.91 for 17 sensitive and 25 resistant cell lines), and hematopoietic tumors (BA = 0.94 for 16 sensitive and 13 resistant cell lines). In contrast, performance for skin cancers (melanomas) reached only a BA of 0.5 for 15 sensitive and 2 resistant cell lines. In the case of AZD6244 (selumetinib, another MEK inhibitor approved for neurofibromatosis type I and pediatric neurofibromas [41]), the largest performance was found for breast tumors (BA = 0.96 for 1 sensitive and 12 resistant cell lines), while performance was much more modest for other cancer types. Classifiers for Paclitaxel showed remarkable performance on ovarian cancer (BA = 0.88 for 4 sensitive and 7 resistant cell lines), a cancer type for which this drug is often part of the first-line treatment [42], and melanoma (BA = 0.82 for 5 sensitive and 10 resistant cell lines), although this latter cancer type is more rarely treated with cytotoxic compounds. Other notable large performances are the ones of two classifiers on pancreatic cell lines: ZD-6474 (vandetanib [43], a VEGFR/EGFR inhibitor) scoring BA = 0.83 for 5 sensitive and 9 resistant cell lines and sorafenib [44], a large-spectrum kinase inhibitor (BA = 0.96 for 6 sensitive and 9 resistant cell lines), the RAF/VEGFR2 inhibitor RAF265 [45] for ovarian cancer (BA = 0.85 for 9 sensitive and 2 resistant cell lines), and the EGFR inhibitor Erlotinib [46] for breast cancer (BA = 0.85 for 5 sensitive and 10 negative cell lines). Supplementary Table S3 shows the performance of the classifiers for all drugs.

### Most important features point to known and new biomarkers

Then, we retrieved the feature importances of the underlying first-level models, or the absolute weights in the case of regression-based algorithms, and computed the average rank of each feature across the 100 sub-folds, separately for each compound.

Our analysis of the importance of the individual features in the different omic-specific datasets indicated that many alterations, including expression of specific genes or phosphoproteins, was reliably utilized by the different first-level algorithms to build their predictions. Independently for each compound and each omic type, we ranked the features according to their importance, which we calculated either, for tree-based algorithms, as the proportion of internal nodes using this feature, or in the case of regression-based algorithms, as the absolute value of the coefficients. We collected these ranks over the 100-folds of the cross-validation scheme.

For Panobinostat, the largest contributions were from the support vector machine (SVM) and logistic classifiers, trained on transcriptomics data (Fig. 3). These classifiers ranked the same four transcripts as the most informative features: *AC138623*.1 (*ZNF141* pseudogene), *AC011242*.6 (a pseudogene transcribed from the reverse strand of the *PLEKHH2* gene), *ZNF215* [47], and *SFMBT2* [48]. The same four features were also picked by the RFC algorithm.

The main contributing algorithms and dataset were the same for paclitaxel, and both SVM and logistic models pointed to a high importance of *LEPREL2* [49] and *MAGEA6* [50], as well as *SLFN11* [51] and *RCOR2* [52, 53].

The main contributions for irinotecan were the AdaBoost and Extra-Trees algorithms (Fig. 3), trained on the transcriptomic data. These, as well as other algorithms trained on the same dataset, highlighted *SLFN11*, *hnRNPA1* [54], *hnRNPCP1*, *DAAM1* [55], as well as two pseudogenes: AC008427.2, also called *MFFP2*, and *RP11-177C12.1*. In the case of Lapatinib, the most contributing datasets were transcriptomics and proteomics, analyzed with SVC (or SVM) and extra-trees, respectively. *GPX3*, *DYRK3*, *ADORA1*, and *STYL1* were among the low-ranking transcriptomics features, while analysis of the proteomic features pointed to Claudin7, E-Cadherin, and Rab25. Notably, most algorithms recovered either EGFR/HER1 or HER2 among their most important features.

Erlotinib appeared as the exception, in having the proteomics as the top-contributing dataset, paired with the Elastic Net algorithm. The most important features in this case appear to be P-Cadherin, EGFR, as well as Shc_pY317 and RSK1-2-3.

For mirdametinib (PD-0325901), the main contribution came from the transcriptomics dataset, through the Elastic Net algorithm. The features with the lowest average rank were *ETV4* and *ETV5*, as well as *SPRY2* and *TOR4A*. We also noted the presence of *CMTM7* among the features consistently ranked low by several algorithms.

In the case of selumetinib (AZD6244), the main contribution was the logistic algorithm, trained on the transcriptomics dataset. The most important feature in this dataset-algorithm pair, as well as in others, appears to be *CMTM7*, as well as *ETV4*, *S100A4*, *SPRY2*/4, and *TRPV2*.

Furthermore, among the top predictors for the other 16 classifiers with inferior performance, we noticed that a number of genes in the transcriptomics datasets were consistently picked up by various algorithms and seemed to be correlated with response, for a variety of compounds. These genes are *MAGEA6*, *NQO1*, and *LEPREL2*, already mentioned, as well as *FAM21B* and *PTEN* for sorafenib, *HERC5* and *CHRNB1* for RAF265, and *SIAH3* for AEW541. PLX4720 (a BRAF inhibitor related to vemurafenib) was the only compound for which the genomic information was the most informative. Unsurprisingly, the BRAFV600E mutation was consistently the feature with the lowest rank for this compound. In the case of PHA665752 and AZD5030, the main contribution to the final classifier were from the miRNA dataset and evidenced the low rank of several microRNAs: miR130a, let-7c, miR1307, miR425, miR222, miR223, and miR34a, among others. The classifiers for Nutlin-3 relied mostly on the proteomics dataset and the Elastic Net or logistic algorithms, and pointed to Bax, VAV1, Annexin1 and p21 as top features. In addition, predictions for nilotinib and PF2341066, of intermediate performance, relied mostly on the cell type, and valued the hematopoietic origin of the tumor cells as the most important factor to predict chemosensitivity.

Finally, we noticed that long non-coding RNAs frequently appeared among the top 50 features retrieved by most algorithms in the transcriptomics database. While these regulatory nucleic acids have received increasing attention recently for their role in tumorigenesis and cancer progression [56], they are still largely understudied. Their presence in our results indicates that they are likely to play a role in the mechanisms underlying sensitivity and resistance in many cases.

We compiled the main predictors of sensitivity discovered by our method in Table 1.

**Table 1:** Summary table of the predictive features evidenced by our modeling study and their relevance to cancer mechanisms

Drug	Clinical trial (phase)	Recommended for cancer type	Biomarker	Functional pathways	Link to resistance	Ref resistance	Ref resistance 2	Ref resistance 3
PD-0325901 (mirdametinib)	Ref Weiss 2021 (phase 2); NCT05054374 (phase 1b/2a)	Neurofibromatosis type-1 associated plexiform neurofibromas (2020)	ETV4	MEK	Binds the MYC enhancers and contributes to both transformation and cellular motility in PC3 prostate cancer cells	Hollenhorst 2010	Zhang 2011	Yuan 2014
			ETV5	MEK	Mediates cancer metastasis, proliferation, oxidative stress response, and drug resistance	Wei 2023		
			SPRY2	MEK	Role in progression of breast and vulvar cancer	Mamoor 2021	Massoumi-Moghaddam 2015	
			DUSP6	MEK	ERK1/2	Xiao 2021		
			CMTM7	PD-L1	High expression means indicates higher sensitivity to immunotherapy	Jiang 2022		
AZD6244 (selumetinib)	Ref Gross 2023; NCT01362803 (Phase 1b/2a)	Neurofibromatosis type-1 associated plexiform neurofibromas (2020)	SPRY2	MEK	Role in progression of breast and vulvar cancer	Mamoor 2021	Massoumi-Moghaddam 2015	
			SPRY4	MEK	SPRY4-IT1 linked to HIF1a and ABC transporters	Zheng 2020		
			ETV4	MEK	Binds the MYC enhancers and contributes to both transformation and cellular motility in PC3 prostate cancer cells	Hollenhorst 2010	Zhang 2011	Yuan 2014
Paclitaxel	Ref Dovehauer 1997	Ovarian (1992); Breast (1994); Lung (1999) (+Pt-based); Kaposi’s sarcoma (1997) (+doxorubicin)	SLFN11	DDR, JAK/STAT	Protects tumors from DNA-damaging agents and predicts response to chemotherapy across several cancers	Willis 2021	Zoppoli 2012	Kagami 2020
			MAGEA6	CC, apoptosis, immune	Expression is cancer-specific and linked to resistance to 5-FU and arsenite	Colemon 2020		
			GPX2	Oxidative stress, DDR	GPX2 overexpression increases the tolerance of cell lines to cisplatin	Wu 2021		
Panobinostat	Ref Laubach 2015; trial Wolf 2011	Myeloma (2007) (+bortezomid + DMX)	TNFRSF12A	TNF	Promotes survival via NFKB and upregulation of Bcl-2 proteins	Whitsett 2014		
			AJUBA	Wnt, RAS/ERK, JAK/STAT, Hippo	Induces YAP-mediated resistance to cisplatin in OSCC	Yoshikawa 2015		
			ZNF215	PTEN/AKT	Expression negatively correlates with survival in patients with AML	Yang 2021		
			RP11-7F17.7	ERK/MAPK	/			
			AC138623.1	?	/			
			AC011242.6	?	/			

(*Continued*)

**Table 1:** Continued

Drug	Clinical trial (phase)	Recommended for cancer type	Biomarker	Functional pathways	Link to resistance	Ref resistance	Ref resistance 2	Ref resistance 3
Irinotecan	Ref Fuchs 2006	Colorectal metastatic (+5-FU) (1996); pancreas (+FOLFININOX) (2005)	SLFN11	DDR, JAK/STAT	Protects tumors from DNA-damaging agents and predicts response to chemotherapy across several cancers	Willis 2021	Zoppoli 2012	Kagami 2020
			RP11-434O22.1	?	/			
			HNRNPA1	RNA Metabolism	Increases response to AR inhibitors in prostate cancer	Zhang 2021		
			KHDC1	AKT, Bcl2	Inhibits apoptosis in head–neck carcinoma	Zhang 2022		
Erlotinib	Ref Cohen 2005	NSCLC (EGFR) (2004); pancreas (2005) (+gemcitabine)	RP11-902B17.1	?	/			
			RP11-47I22.1	?	/			
			IFI27	Immune	Associated with response to immunotherapy	Huang 2023		
			CORO2A	Actin	Associated with multiple clinical factors including survival and immune infiltration	Xie 2023		
			TSTD1	Hypoxia	Overexpression is linked with poor response in breast cancer	Ansar 2022		
Lapatinib	EGF100151 (phase 3)	Breast (2007) (+capecitabine or letrozole)	RP11-902B17.1	?	/			
			RP11-47I22.1	?	/			
			DYRK3	Mitosis	Role in development of OSCC and its resistance to radiotherapy	Huang 2023		
			SYTL1	Ca++	Overexpressed in various cancers, associated with poor prognosis	Suo 2022		
			GPX3	Oxidative stress, DDR	Downregulation increases sensitivity to platinum-based agents	Hu 2023		
			ADORA1	Adenosine receptor	Correlates with immune infiltrates in thyroid carcinoma	Lin 2021		
			GPR135	Metabolite-sensing	Involved in gastric cancer progression	Li 2022		

### Comparisons with single data types

**Figure 5:**
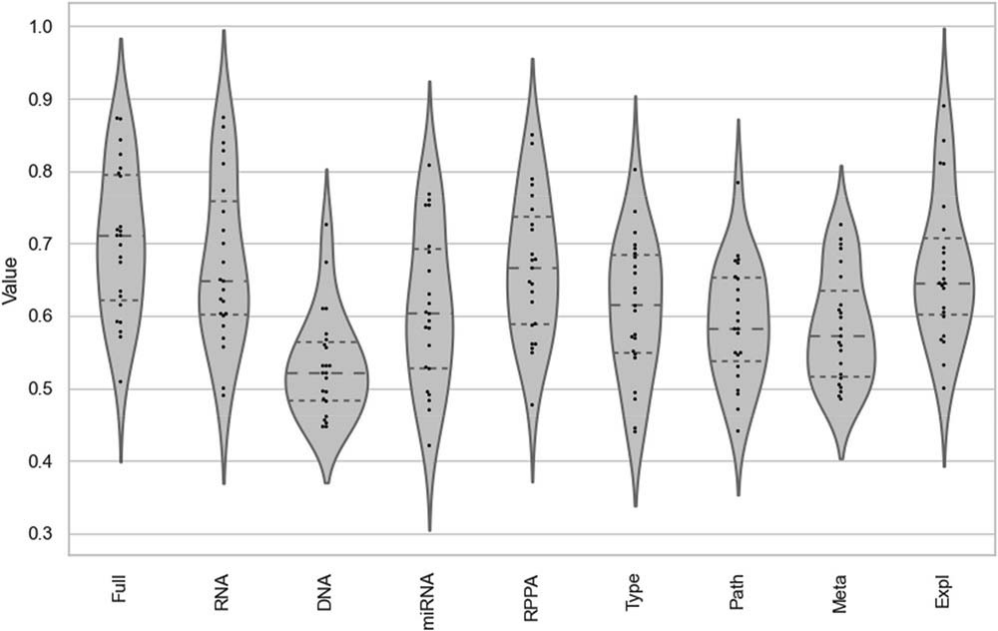
Violin plot showing the distribution of drug-specific predictive models trained on the different subsets of the CCLE dataset. Path: pathway activities; Meta: metabolomic; Expl: explainable models.

We applied our modeling pipeline to the individual parts of the CCLE dataset, to compare the performance of stacked classifiers drawing from the complete dataset with the performance of the same procedure but considering a single dataset at the time (Table S3). In general, we observe a small improvement of the performance when considering multiple datasets, however it is not the case for all drugs. The transcriptomics data alone is often enough to obtain accuracies that are comparable, or even slightly superior to the full multi-omic dataset (Fig. 5). For paclitaxel and irinotecan, for example, the models trained only on the transcriptomic data were slightly more performant than the ones trained on the full dataset. For TAE684 and ZD-6474, it was the RPPA dataset that performed slightly better, and in the cases of AEW541 and PF2341066, cancer type only could resume and even surpass the performance of the full model.

### Explainable models can capture most of the predictivity of ensembles

**Figure 6:**
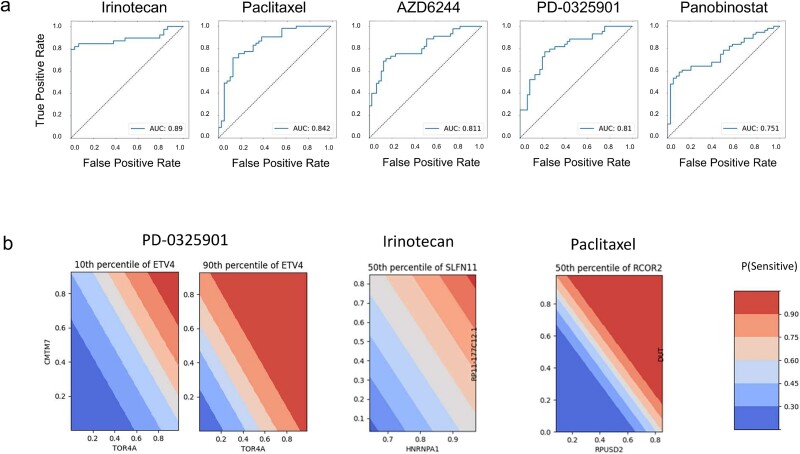
(a) ROC curves for the five drug-specific explainable models with AUROC >0.75; (b) Decision thresholds for three example drugs.

In order to build interpretable, useable predictive models, we attempted to use only the dataset with the highest predictivity alone (transcriptomics) and reduced the number of features to three. Figure 6a shows the ROC curves for five best of these slim models, for which AUROC >0.75. Figures 6b show the 2D partial dependency plots for two example drugs, for the respective models. Supplementary Fig. S2 shows the ROC curves for each drug’s best model. It can be noted that the five drugs with the best results [irinotecan, paclitaxel, panobinostat, AZD6244 (selumetinib), PD-0325901 (mirdametinib)] were already individualized during the previous analysis. The partial dependency plots for irinotecan (Fig. 5b, middle) shows that, as the level of expression of each of the three predictor genes (HNRNPA1, RP11-177C12.1 and SLFN11) increases, so does the predicted sensitivity of the cell lines. A similar reasoning can be made for paclitaxel (Fig. 5, right).

Furthermore, we assessed the generalizability of this approach by testing the models on the GDSC database [38]. Using gene expression data from 967 cell lines and AUC values for four drugs overlapping with the CCLE models (irinotecan, paclitaxel, AZD6244, and PD-0325901), we applied the same predictors. Our results suggest a strong applicability of our modeling strategy across databases, supporting the feasibility of constructing explainable models based on the expression of a small set of genes, regardless of the dataset (Supplementary Table S4). Further work is needed to develop these models across more databases and develop models that can be applied in a clinical setting.

We also tested the influence of the data splitting strategy, by comparing our original labeling with two alternatives: one where only the first and last quartiles are labeled and the remaining two are kept out, and one where nearly all the samples are labeled, leaving only 5% of the samples in the unlabeled category. We tested our seven best models in a 5-times-5-times nested cross-validation scheme with these three strategies. Our results (Supplementary Fig. S2) indicate that the original strategy is adequate, as both alternatives lead to inferior results in terms of AUROC.

Finally, we tested the hypothesis that a simpler second-level algorithm would be either superior or equivalent to the random forest integrator we used [57]. To do so, we retested our seven best models in a 5-times-5-times nested cross-validation scheme, this time comparing random forest with logistic regression (Supplementary Fig. S3). Our results indicate a near-perfect correspondence of the ROC curves, suggesting that while our random forest approach is adequate, simpler models are able to achieve the same performance in integrating the predictions of first-level omics.

## Discussion

Here we describe an analysis pipeline, comprising an ensemble learner (random forests) trained on the predictions of a set of machine-learning algorithms, themselves trained separately on the various omic datasets of the CCLE database. We separated the cell lines, for each of the 23 compounds, into three equal-sized categories: sensitive, intermediate, and resistant, and applied nested cross-validation to classify sensitive *versus* resistant cell lines. Our results indicate that for seven compounds (three cytotoxic: paclitaxel, irinotecan, and panobinostat; four targeted: mirdametinib, selumetinib, erlotinib, and lapatinib) we can predict the position of cell lines within these two categories, across cancer types, with remarkable performance. Nevertheless, the performance of our classifiers varied with cell type: better results were obtained for cancer types for which many cell lines are present in the CCLE database (e.g. lung carcinoma and colorectal carcinoma) while performance was less convincing for a number of other cancer types showing fewer representative cell lines in the CCLE database.

Contrary to our expectations, ensembling and late-stage integration of predictions only moderately improved the performance compared to the single-omic models. In most cases, compared to the full model trained on the complete multi-omic dataset, models trained on a single data type obtained similar performance. This seems to show that the predictive signals are redundant across omic types, and do not necessarily synergize or complement each other. The transcriptomics datasets were the data type that contained the most information, which can now be re-interpreted in a more clinical application context.

For example, genes identified by our improved analysis belong to different families and pathways, and while the function of several have already been published previously, most of them had never been targeted in a clinical context. For example, *SLFN11*, a gene coding for a helicase involved in DNA repair, is a known predictor of sensitivity to a wide range of DNA-damaging agents, and has been associated with sensitivity to PARP inhibition [58, 59]. Hence, our study suggests to incorporate this gene into patient diagnosis.

In contrast, *LEPREL2*, plays an important role in collagen chain assembly, and its expression seems to be predictive to resistance to the inhibitor of spindle formation paclitaxel. *LEPREL2* has previously been identified, together with *TGFBI*, as part of a hub of genes controlling the response to 5-fluorouracil-based chemotherapy in colorectal cancer, although the level of expression of this gene was not itself significantly different between resistant and sensitive cell lines [60]. A more detailed analysis in a cancer-specific context taking a whole hub of genes into account might help for patients’ stratification here.

For example, MEK inhibitors, selumetinib and mirdametinib, present partially similar profiles of predictors: for these two compounds, expression of ETV4, a transcription factor involved in the regulation of transcription by RNA polymerase II, as well as SPRY1/2/4, seem to be associated with sensitivity. ETV4 has been associated with a number of cancers [61, 62]. Notably, ETV4 was recently correlated with poor survival, as well as with immune cell infiltration, tumor heterogeneity and stemness in a pan-cancer cohort in TCGA [63]. The SPRY family of genes encodes a number of proteins involved in the negative regulation of growth signaling [64]. More importantly, the long non-coding RNA SPRY1-IT1 has been associated, both positively and negatively, with proliferation and metastasis in breast, liver, and gastric cancers [65–67]. The role of this long non-coding RNA in relation to cancer has recently been partially elucidated, revealing functional interactions with several cancer-associated pathways, notably HIF-1alpha, NFκB, and the MAPK/PI3K axis [68]. SPRY2 has been associated with cancer progression in particular in breast cancers and melanomas [69].

In the case of the topoisomerase-inhibitor irinotecan, two genes seem to be highly predictive: HNRNPA1, an abundant and ubiquitously expressed member of the hnRNP family of heterogeneous-nuclear-ribonucleoproteins, and CMTM7, or CKLF-like MARVEL transmembrane domain-containing protein 7, a gene involved in various cellular processes, including immune regulation and cancer development. HNRNPA1 is known to interact with, and regulate the expression and translation of, key factors of tumorigenesis, in particular apoptosis, cell cycle, and telomere length maintenance [54]. Strikingly, while this gene is overexpressed in a number of cancers, it has not, to our knowledge, been associated with sensitivity to any compounds. CMTM7 has been determined to be downregulated in various cancers, and its overexpression inhibits cell proliferation and tumor formation. For these reasons, it could potentially function as a biomarker [70]. Interestingly, TNFRSF12A, a member of the Tumor Necrosis Factor receptor superfamily, was found associated, in our analyses, with sensitivity to the HDAC-inhibitor panobinostat.

Furthermore, we designed predictive models based on subsets of predictive transcriptomic features. In a number of cases, our results indicate that the sensitivity of cell lines to antineoplastic agents, either cytotoxic or targeted, can be predicted with a high degree of accuracy and specificity. We propose that these models, which only require the measurement of the level of expression of a small number of genes, could be useful in the assessment and stratification of patients, and could be instrumental in the progress toward individualized cancer treatment. For example, our model for irinotecan, which only relies on a linear model of three RNA species (HNRNPA1, RP11-177C12.1 and SLFN11), is able to pick the most sensitive cell lines across cancer types, potentially rendering this drug useful in patients presenting with cancers for which this drug is not part of the standard treatment. Similarly, our models for selumetinib and mirdametinib are able to segregate sensitive from resistant cell lines, including in cancer types which usually do not present any of the known alterations of the MAPK pathway and for which MEK inhibitors are usually not recommended. Our models for paclitaxel and panobinostat obtain similarly interesting performances.

Conclusively, our results indicate that large-scale analyses of cancer cell line repositories are useful to retrieve relationships between resistance to anti-cancer drugs and gene expression profiles. Our pipeline exploits the major signals of the dataset by focusing on the most extreme functional differences. By considering the ActArea, which considers the entirety of the drug-response curve, as a target instead of the IC50, we were able to focus on integrated functional response. Our pipeline uses multiple cross-validation steps, which helps in balancing the biases potentially introduced by the relatively small number of samples in biological databases compared with the large number of features. Many of the predictors recovered by our stacked modeling fall in line with previously published results [71, 72]. In addition, we individualized a number of species, some of which understudied like long non-coding RNAs, which seem to play a role in cancer development, and recommend that further research focuses on these targets to shed light on their involvement in the various processes of carcinogenesis. Hence, our findings strongly recommend to extend patient stratification beyond genomic profiling to transcriptomic analysis of at least a subset of cancer specific (or drug specific?) candidate genes, paving the avenue to individualized cancer therapy/treatment.

This study has a number of important limitations. When evaluating the predictive performance of our models, it is important to remember that a third of the cell lines (not necessarily the same across compounds) have been excluded from the dataset, as they displayed an intermediate level of drug responsiveness which could decrease the ability of our models to form accurate predictions on the more extreme phenotypes. Therefore, a strong correlation between our predictions and the measured sensitivity of cell lines to the tested compounds exists, this ‘intermediate’ class of cell lines is likely to display a mix of molecular characteristics from both sensitive and resistant cells, or could display its own molecular characteristics, which we did not explicitly study. Future works should focus on addressing this issue. In addition, the genetic make-up of the cell lines of the CCLE database does not necessarily represent accurately the variability in a large human population. Ideally, this should be accounted for when designing future clinical studies pertaining to the evaluation of genetic markers. It has also been noted previously that the ActArea, although arguably a better indicator of drug sensitivity than the IC50, is harder to learn for predictive models [73].

The main takeaway of our study is that, contrary to expectations, the transcriptomic dataset is nearly always a better feature set to build predictors of sensitivity than other omics, notably genomics. This can be explained by several factors, notably the continuous nature of RNA sequencing data (in contrast to Boolean genomic information), the more direct link with proteins which are the primary effectors and mediators of the effects of various drugs, and the fact that RNA analysis better reflect the variability in gene expression among cells carrying the same genetic mutation. In addition, transcriptomic data is able to capture the effects of post-transcriptional modifications, possibly impacting drug response. This is in line with the results of recent clinical trials, showing moderate but tangible improvements in the clinical outcome of patients following integration of gene expression analysis in therapeutic decision-making [74, 75], and provides additional arguments for biomarker-based treatment strategies [76, 77].

The second conclusion is that the accuracy of sensitivity predictions for cell lines varies greatly depending on the drug studied. This can be explained by the presence or absence of adequate predictive signals within the dataset, owing to the specific mechanism of action of the compound. Still, we observe important differences in the performance of our models for drugs with similar clinical profiles, for example DNA-damaging agents, or MEK inhibitors. This observation emphasizes the specificities of different drugs belonging to the same class and the necessity of assessing a large array of compounds.

Lastly, we identified a series of genes which expression bare a predictive potential of sensitivity in our dataset for multiple cancer types, and provided examples showing that explainable models using limited set of maximum three transcriptomic markers can retain most of the predictive power of large ensembles. We propose that future research focus on designing and validating such minimalistic models with the possibility to incorporate them as decision tools for clinicians.

**Key Points**

- We developed models predictive of the sensitivity of cell lines to anti-cancer drugs.
- Late-stage integration of multiple models built from single omic layers did not improve significantly the accuracy of the models compared with single-omic models.
- We identify SLFN11, ETV4, HNRNPA1, and CMTM7 as promising markers of drug sensitivity in a number of common cancers.
- Long non-coding RNAs have the potential to be used as predictors of cancer sensitivity.
- Transcriptomic information is more predictive than other omic layers in most investigated cases.

## Supplementary Material

REV_FigS1_ROC23compounds_bbae567

REV_FigS2_ComparisonSplits_bbae567

REV_FigS3_Figure_Integrator_bbae567

REV_ML_CCLE_supplMethods+TableS1_bbae567

REV_TableS2_SuppTableStats_bbae567

REV_TableS3_Perfs_bbae567

REV_TableS4_CCLEGDSC_bbae567

Figure_and_table_caption_bbae567

## Data Availability

All data associated with this publication, as well as necessary Python code to reproduce the results, are available at the following address: https://github.com/sysbiolux/DeepOncoAI.
