# Supplementary figures and images for: Model ensembling as a tool to form interpretable multi-omic predictors of cancer pharmacosensitivity
Source: Brief Bioinform. 2024 Nov 4;25(6):bbae567. doi: 10.1093/bib/bbae567 (PMC11532660; doi:10.1093/bib/bbae567)

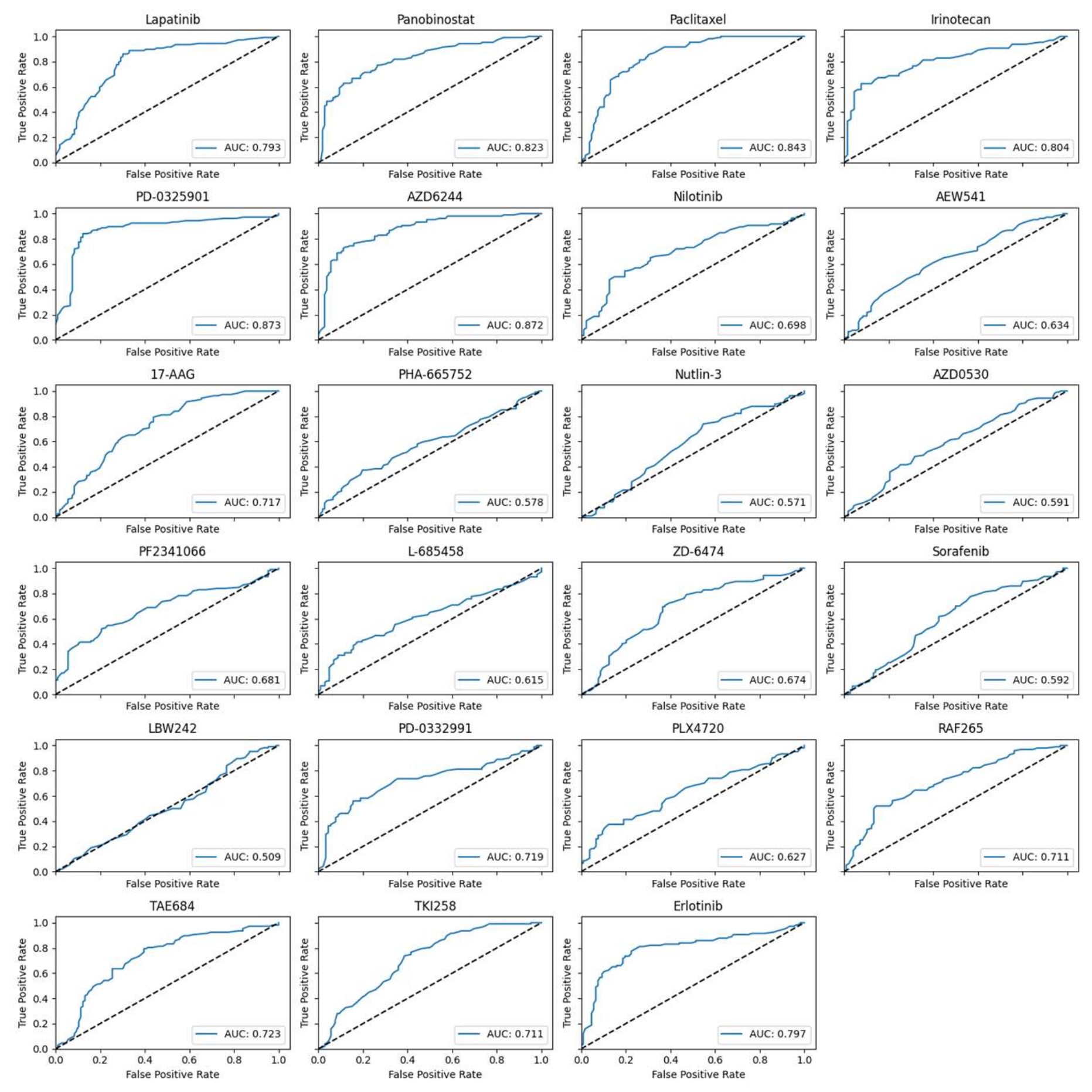

Supplement: REV_FigS1_ROC23compounds_bbae567 [file rev_figs1_roc23compounds_bbae567.jpeg]

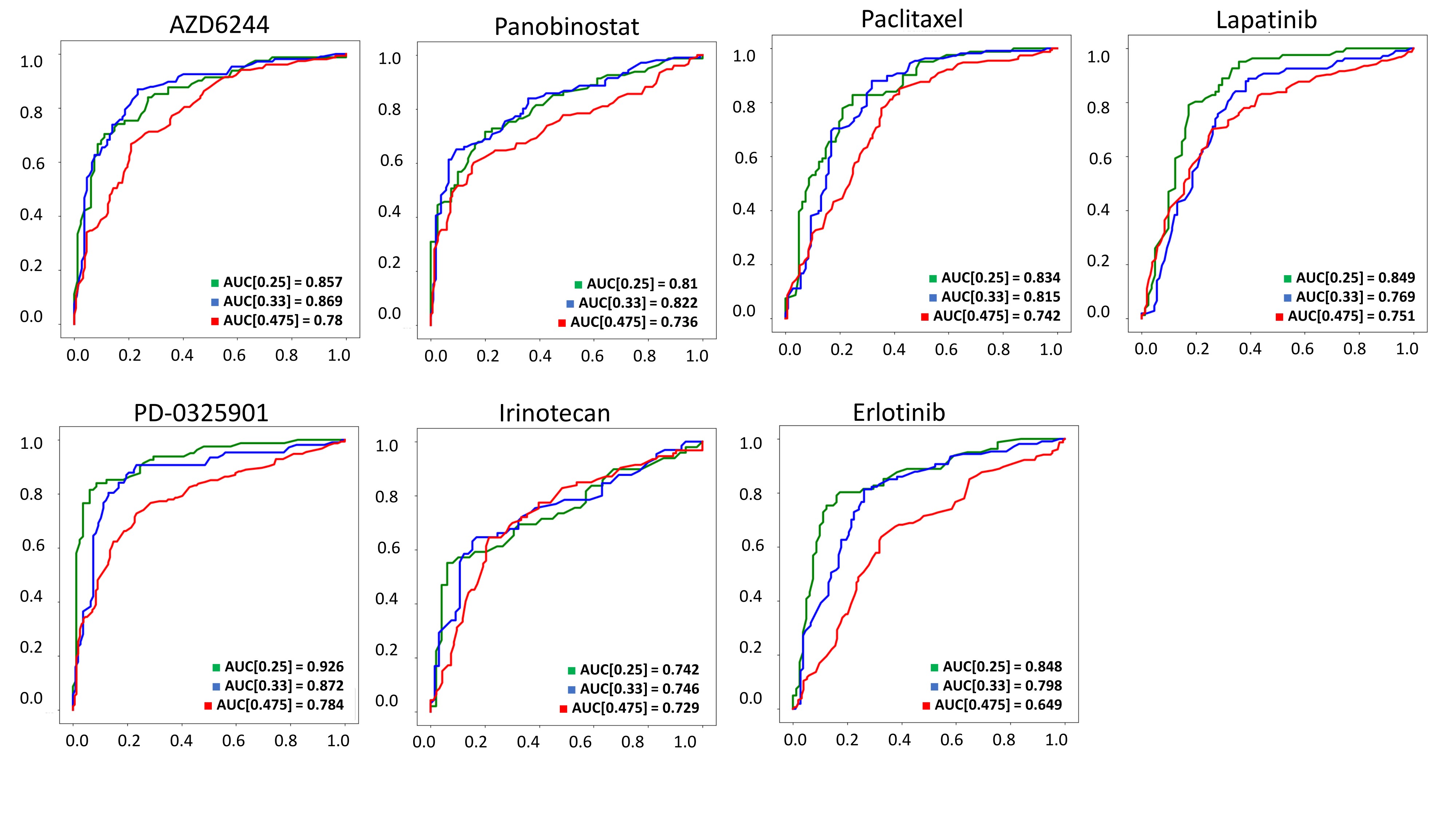

Supplement: REV_FigS2_ComparisonSplits_bbae567 [file rev_figs2_comparisonsplits_bbae567.jpeg]

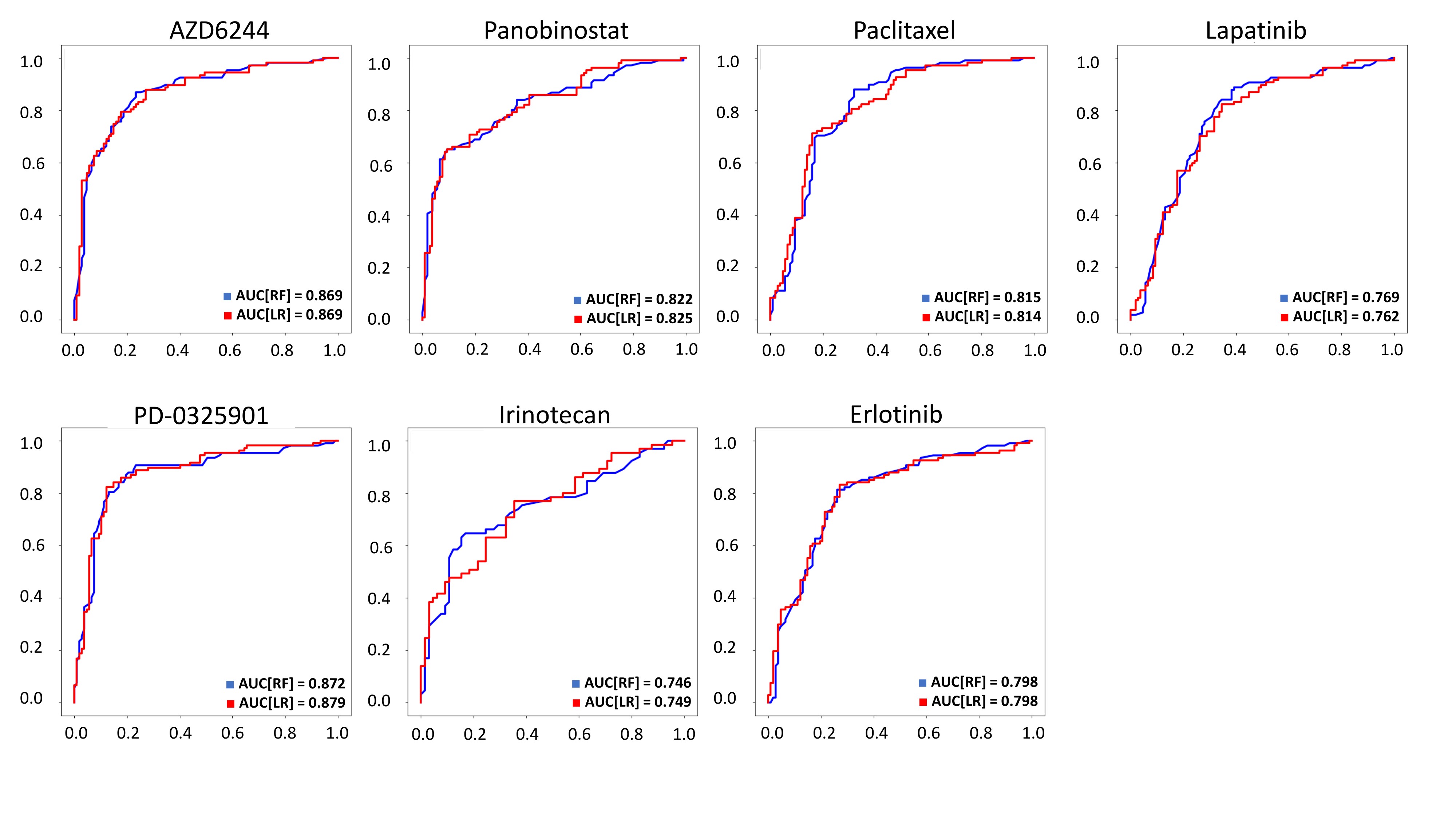

Supplement: REV_FigS3_Figure_Integrator_bbae567 [file rev_figs3_figure_integrator_bbae567.jpeg]
