# Supplementary material for: Model ensembling as a tool to form interpretable multi-omic predictors of cancer pharmacosensitivity
Source: Brief Bioinform. 2024 Nov 4;25(6):bbae567. doi: 10.1093/bib/bbae567 (PMC11532660; doi:10.1093/bib/bbae567)
Supplement: REV_ML_CCLE_supplMethods+TableS1_bbae567 [file rev_ml_ccle_supplmethods+tables1_bbae567.docx]

Model Ensembling as a Tool to Form Interpretable Multi-Omic Predictors of Cancer Pharmacosensitivity

Sébastien De Landtsheer^1^, Apurva Badkas^1^, Dagmar Kulms^2, 3^, Thomas Sauter^1^*

^1^Department of Life Sciences and Medicine, University of Luxembourg, Belvaux, Luxembourg

^2^Experimental Dermatology, Department of Dermatology, TU-Dresden, 01307 Dresden, Germany

^3^National Center for Tumor Diseases, TU-Dresden, 01307 Dresden, Germany

*: Corresponding author: [thomas.sauter@uni.lu](mailto:thomas.sauter@uni.lu)

Supplementary Methods

*Random Forests* is a learning method, in which multiple decision trees are constructed independently from bootstrapped instances of the dataset and a random selection of the original features. The individual trees are grown using the Gini impurity at each split, and the prediction of the random forest for a specific instance is the mode (for classification problems) or the average (for regression problems) of the predictions of every decision tree in the ensemble [1]. We used forests of 100 trees, selecting for each one a fraction $\surd(F)$ of the F features.

*AdaBoost* is a learning method, related to random forests, in which an ensemble of weak learners (in our case decision stumps, which are decision trees of depth 1) is built sequentially in such a way that at each step, the subsequent learner is trained on the original dataset but the weights of correctly and incorrectly classified samples are modified to help the new learner focus more on the errors of the previous learners [2]. This ‘boosted’ learner improves the classification performance of the ensemble, and predictions for specific instances are computed as the mode or average of the predictions of the constituent learners. We used this algorithm with 50 decision stumps and a learning rate of 1.

*XGBoost* is also a learning method based on boosted learners, and includes multiple features, notably automated handling of missing data, stochastic and regularized gradient boosting, and overall better memory and CPU utilization, resulting in this algorithm being one of the best out-of-the-box tools for machine-learning [3]. We used XGBoost with a learning rate of 0.3, a maximum depth of the constituent trees of 6, no subsampling of samples or features, and values of 0 and 1 for the L1 and L2 regularization terms, respectively.

*ExtraTrees* is another learning algorithm based on boosted learners, with random subsampling of features but without bootstrapping of the samples. In contrast with other boosting algorithms, it chooses the decision threshold at each split at random from the empirical distribution of each feature rather than the value that maximizes information gain [4]. We used this algorithm with 100 constituent trees and a fraction $\sqrt{F}$ of the F features for each tree.

*Logistic regression* is a widely used linear learning algorithm that models the logarithm of the odds of a binary variable as a linear combination of one or more independent predictors. This regressor can be used as a classifier by setting a threshold of probability for class assignment. We used logistic regression with a L2 regularization term of value 1 to limit overfitting.

*Ridge regression* is a variant of logistic regression which does not include a L2 regularization term, but only a L1 regularization term to induce sparsity in the choice of the independent predictors in the case of ill-posed problems, for example in case of multiple collinearities. We used a ridge classifier with a L1 term of 1, and a tolerance of $1\times{10}^{-3}$.

*Elastic Net regression* combines the L1 and L2 regularization terms in a unique optimization problem, thereby aiming for balance between accuracy of the prediction, and sparsity of the predictors as well as their coefficients. We used this classifier with equal weighting of the L1 and L2 penalties and a tolerance of $1\times{10}^{-3}$.

*Support-Vector Machines* are learning algorithms that map, explicitly or implicitly, a set of labeled data to a multi-dimensional space and find the hyperplane with the largest possible margins that separates best the data, as assessed by the Euclidian distance of the closest datapoints to the hyperplane. Such a maximum-margin classifier is guaranteed to minimize the generalization error of the classifier. We used a linear kernel with a L2 regularization value of 1, parameter shrinkage [5], and a tolerance of $1\times{10}^{-3}$.

*k-Nearest-Neighbor* is a learning algorithm where class predictions are made locally from the class membership of a number k of similar labeled examples [6]. It has the appreciable properties of having a single parameter (k) and not requiring any training. We used this algorithm with a fixed k of 5 and used Euclidian distance (Minkowski metric with $p=2$).

In our experiments, we used the AUROC (Area Under the Receiver-Operating Curve) as the optimized metric by all classifiers.

Supplementary Table 1

| Dataset | Sample Completeness threshold | Feature Completeness threshold | Feature Variance threshold | Cross-correlation threshold | Number of features used for modeling |
| --- | --- | --- | --- | --- | --- |
| Transcriptomics | 0.9 | 0.9 | 0.5 | 0.75 | 23398 |
| Genomics | 0.99 | 0.99 | 0.5 | NA | 23135 |
| Proteomics | 0.9 | 0.9 | 0.2 | 0.9 | 165 |
| miRNomics | 0.95 | 0.95 | 0.5 | 0.75 | 631 |
| Metabolomics | 0.95 | 0.95 | 0.5 | 0.75 | 67 |
| Cell types | NA | NA | NA | NA | 23 |
| Pathways | 0.95 | 0.95 | 0 | 0.9 | 11 |
|  |  |  |  |  |  |

Table S1. Characteristics of the data pre-processing steps for each omic type. Sample completeness threshold: samples with a proportion of missing data across features higher than (1 - threshold) were removed. Feature completeness threshold: features with a proportion of missing data across samples higher than (1 - threshold) were removed. Feature variance threshold: variance was calculated for each feature independently across samples and a proportion (threshold) of the most variable features were removed. Cross-correlation threshold: pairwise Pearson correlation coefficients p were computed for all pairs of features within a dataset. For each pair with p > threshold, the feature with the lowest average correlation with the rest of the dataset was retained. NA: not applicable.

Supplementary References:

[1] L. Breiman, “Random Forests,” *Mach. Learn.*, vol. 45, no. 1, pp. 5–32, 2001, doi: 10.1023/A:1010933404324.

[2] Y. Freund and R. E. Schapire, “A Decision-Theoretic Generalization of On-Line Learning and an Application to Boosting,” *J. Comput. Syst. Sci.*, vol. 55, no. 1, pp. 119–139, Aug. 1997, doi: 10.1006/jcss.1997.1504.

[3] T. Chen and C. Guestrin, “XGBoost: A Scalable Tree Boosting System,” in *Proceedings of the 22nd ACM SIGKDD International Conference on Knowledge Discovery and Data Mining*, Aug. 2016, pp. 785–794. doi: 10.1145/2939672.2939785.

[4] P. Geurts, D. Ernst, and L. Wehenkel, “Extremely randomized trees,” *Mach. Learn.*, vol. 63, no. 1, pp. 3–42, Apr. 2006, doi: 10.1007/s10994-006-6226-1.

[5] C.-C. Chang and C.-J. Lin, “LIBSVM: A library for support vector machines,” *ACM Trans. Intell. Syst. Technol.*, vol. 2, no. 3, pp. 1–27, Apr. 2011, doi: 10.1145/1961189.1961199.

[6] Fix, Evelyn, “Discriminatory Analysis, Nonparametric Discrimination: Consistency Properties,” USAF School of Aviation Medicine, Randolph Field, Technical Report 4, 1951.
