# Supplementary material for: Model ensembling as a tool to form interpretable multi-omic predictors of cancer pharmacosensitivity
Source: Brief Bioinform. 2024 Nov 4;25(6):bbae567. doi: 10.1093/bib/bbae567 (PMC11532660; doi:10.1093/bib/bbae567)
Supplement: REV_TableS3_Perfs_bbae567 [file rev_tables3_perfs_bbae567.pdf]

SDL0

| Drug | Total | RNA only | DNA only | miRNA only | RPPA only | TYPE only | PATHWAYS only | META only | Explainable |
|---|---|---|---|---|---|---|---|---|---|
| PD-0325901 | 0.873 | **0.874** | 0.674 | 0.808 | 0.838 | 0.632 | 0.673 | 0.675 | 0.81 |
| AZD6244 | **0.872** | 0.828 | 0.61 | 0.76 | 0.85 | 0.802 | 0.604 | 0.514 | 0.811 |
| Paclitaxel | 0.843 | **0.861** | 0.726 | 0.768 | 0.766 | 0.614 | 0.622 | 0.693 | 0.842 |
| Panobinostat | **0.823** | 0.81 | 0.575 | 0.753 | 0.747 | 0.744 | 0.784 | 0.699 | 0.751 |
| Irinotecan | 0.804 | **0.839** | 0.482 | 0.753 | 0.719 | 0.698 | 0.678 | 0.726 | 0.89 |
| Erlotinib | **0.797** | 0.773 | 0.531 | 0.63 | 0.789 | 0.659 | 0.636 | 0.615 | 0.694 |
| Lapatinib | **0.793** | 0.744 | 0.514 | 0.688 | 0.781 | 0.686 | 0.676 | 0.563 | 0.674 |
| TAE684 | 0.723 | 0.603 | 0.461 | 0.483 | **0.726** | 0.715 | 0.492 | 0.582 | 0.642 |
| PD-0332991 | **0.719** | 0.623 | 0.567 | 0.696 | 0.647 | 0.668 | 0.654 | 0.654 | 0.687 |
| 17-AAG | 0.717 | **0.718** | 0.531 | 0.559 | 0.644 | 0.485 | 0.593 | 0.559 | 0.651 |
| RAF265 | **0.711** | 0.7 | 0.521 | 0.662 | 0.633 | 0.569 | 0.549 | 0.608 | 0.638 |
| TKI258 | **0.711** | 0.65 | 0.56 | 0.584 | 0.619 | 0.494 | 0.549 | 0.489 | 0.665 |
| Nilotinib | 0.698 | 0.648 | 0.456 | 0.593 | 0.666 | 0.681 | 0.683 | **0.706** | 0.605 |
| PF2341066 | 0.681 | 0.674 | 0.531 | 0.605 | 0.678 | **0.693** | 0.582 | 0.572 | 0.599 |
| ZD-6474 | 0.674 | 0.586 | 0.447 | 0.495 | **0.677** | 0.551 | 0.651 | 0.534 | 0.572 |
| AEW541 | 0.634 | 0.62 | 0.495 | 0.583 | 0.561 | **0.638** | 0.546 | 0.552 | 0.645 |
| PLX4720 | **0.627** | 0.603 | 0.61 | 0.527 | 0.549 | 0.607 | 0.582 | 0.519 | 0.611 |
| L-685458 | 0.615 | **0.636** | 0.485 | 0.617 | 0.589 | 0.574 | 0.576 | 0.604 | 0.719 |
| Sorafenib | 0.592 | **0.6** | 0.447 | 0.529 | 0.561 | 0.571 | 0.53 | 0.495 | 0.567 |
| AZD0530 | 0.591 | 0.557 | 0.496 | 0.491 | 0.555 | 0.44 | 0.497 | **0.598** | 0.532 |
| PHA-665752 | 0.578 | 0.569 | 0.521 | **0.603** | 0.587 | 0.542 | 0.517 | 0.505 | 0.564 |
| Nutlin3 | 0.571 | 0.5 | 0.452 | 0.421 | **0.685** | 0.445 | 0.471 | 0.501 | 0.645 |
| LBW242 | 0.509 | 0.49 | **0.556** | 0.47 | 0.477 | 0.547 | 0.441 | 0.485 | 0.5 |

## Slide 8

**SDL0** Table S3

Sébastien DE LANDTSHEER, 2024-07-02T08:29:35.354
