# Supplementary material for: Model ensembling as a tool to form interpretable multi-omic predictors of cancer pharmacosensitivity
Source: Brief Bioinform. 2024 Nov 4;25(6):bbae567. doi: 10.1093/bib/bbae567 (PMC11532660; doi:10.1093/bib/bbae567)
Supplement: Figure_and_table_caption_bbae567 [file figure_and_table_caption_bbae567.docx]

FigS1. Summary ROC curves for the 23 drug-specific models. The jagged curve displays the relationship between sensitivity (True Positive Rate, y-axis) and specificity (False Negative Rate, x-axis) for different decision thresholds. The dashed line shows the theoretical performance of a random model.

TableS3. Performances (Area under the ROC curve) for the different drug models, either with all available omic types (Total) or with single omic layers. The best performance for each model is indicated by a bold value.

TableS2. Summary table of the performance of individual models for each cancer type. tpn: number of true positives; tnn: number of true negatives; fpn: number of false positives; fnn: number of false negatives; sens: sensitivity; spec: specificity; prec: precision; npv: negative predictive value; f1: F1-score; ba: balanced accuracy; mcc: Mathew's correlation coefficient; w_mcc: weighted Mathew's correlation coefficient.

TableS1. Characteristics of the data pre-processing steps for each omic type. Sample completeness threshold: samples with a proportion of missing data across features higher than (1 - threshold) were removed. Feature completeness threshold: features with a proportion of missing data across samples higher than (1 - threshold) were removed. Feature variance threshold: variance was calculated for each feature independently across samples and a proportion (threshold) of the most variable features were removed. Cross-correlation threshold: pairwise Pearson correlation coefficients p were computed for all pairs of features within a dataset. For each pair with p > threshold, the feature with the lowest average correlation with the rest of the dataset was retained. NA: not applicable.

Table1. Summary table of the predictive features evidenced by our modeling study, and their relevance to cancer mechanisms.

TableS4. Performances (Areas under the ROC curve) for the models trained on the CCLE transcriptomics (CCLE) versus the GDSC database, for four example explainable models.

FigS2. ROC curves comparing different labeling thresholds. Resistant, intermediate, and sensitive cell lines were assigned based on either the [0.33-0.33-0.33] quantiles separation presented in the main paper, or a more conservative one [0.25-0.5-0.25] or a more inclusive one [0.475-0.05-0.475].

FigS3. ROC curves comparing the Random Forest (RF) integrator presented in the main paper with the simpler Logistic Regression (LR) integrator. Both models used the same first-level algorithms predictions.
